# Should I Change Anticoagulane in Veno-Venous ECMO?

**DOI:** 10.4274/TJAR.2025.241745

**Published:** 2025-07-24

**Authors:** Rabia Yılmaz, Murat Arslan, Deniz Özel Bilgi, Zafer Çukurova

**Affiliations:** 1 University of Health Sciences Türkiye Bakırköy Dr. Sadi Konuk Training and Research Hospital, Clinic of Anaesthesiology and Reanimation Critical Care, İstanbul, Türkiye

**Keywords:** Anticoagulant, bivaluridin, bleeding, ECMO, thrombosis

## Abstract

**Objective:**

Due to a lack of high-quality data to guide anticoagulation therapy in extracorporeal membrane oxygenation (ECMO) patients, there is significant variation in practice among centers. We aimed to investigate the safety, anticoagulation efficacy, and cost-effectiveness of using bivaluridine as a primary anticoagulant without unfractionated heparin (UFH) in ECMO patients.

**Methods:**

The study population included patients undergoing Veno-Venous ECMO for acute respiratory distress syndrome. A total of 56 patients were evaluated, 25 were on UFH and 31 were on bivalirudin.

**Results:**

There was no significant difference between the time to reach the target activated partial thromboplastin time (aPTT) interval [6 (3.5-11) UFH, 9 (4-19) bivalirudin, *P*=0.287]. There was no significant difference between the percentage of time spent in the target aPTT interval (61.48±14.72 UFH, 62.65±11.99 bivaluridine, *P*=0.745). The median amount of erythrocyte suspension replacement (12.04±8.01; 7.9±4.71; *P*=0.028) and the median amount of fresh frozen plasma replacement [4 (2-6); 1 (0-4); *P*=0.001] were higher in the UFH group than in the bivaluridine group. The cost was lower in the UFH group compared to the bivalirudin group [$38.1 (13.5-48.7); $463.7 (194.3-819.8); *P *< 0.001].

**Conclusion:**

The use of bivaluridine as a primary anticoagulant does not lead to any decrease in anticoagulant efficacy.

**Main Points**

• The primary result of our study is that the use of bivaluridine as a primary anticoagulant in extracorporeal membrane oxygenation (ECMO) does not cause any decrease in anticoagulant effectiveness.

• In our study, evaluating the number of patients with thrombus and bleeding alongside the total amount of replaced blood products suggests that the use of bivaluridine is more suitable than the use of unfractionated heparin (UFH) in patients undergoing Veno-Venous ECMO. In the bivaluridine group, where all patients had coronavirus disease-2019 (COVID-19), no difference occurred in thrombotic events despite the prothrombotic effect of COVID-19.

• The total amount of replaced erythrocyte solution and total amount of fresh frozen plasma solution was statistically significantly higher in the UFH group than in the bivalirudin group.

• The cost of providing 15-day anticoagulation in the UFH group is considerably lower than in the bivalirudin group, which is an advantage for the use of UFH.

## Introduction

Extracorporeal membrane oxygenation (ECMO) is an invasive and last-resort treatment for circulatory and respiratory failure. Lack of high-quality data to guide anticoagulation management in ECMO patients results in marked practice variability among centers. Systemic anticoagulation is still a necessity in ECMO patients to prevent the development of ECMO circuit thrombosis and deep vein thrombosis. Unfortunately, the ideal pharmacologic anticoagulant agent remains unclear. While the risk of thrombosis is reduced with all anticoagulant therapies, the risk of bleeding increases. Intermittent blood product replacement is required to replace the loss of blood cells and clotting factors due to both bleeding and extracorporeal circulation.

The widely used anticoagulant in ECMO patients is heparin, which contributes significantly to the development of ECMO with its discovery.^1, 2, 3^ Unfractionated heparin (UFH) is a glycosaminoglycan that binds to antithrombin (AT) to produce a 1000-fold increase in AT inhibition of thrombin, factor Xa, factor XIIa, and factor IXa.^4^ The half-life of UFH in adult patients is 60-90 minutes.^1^ UFH has the advantages of being cheap, accessible, and having an antidote (protamine). In addition to binding to AT, UFH binds to circulating plasma proteins, endothelial cells, and macrophages, which changes the pharmacokinetics of the drug and makes dose adjustment difficult. Although heparin is the most commonly used in clinical applications, heparin resistance is a major concern in ECMO. It is defined as a situation where the ability of heparin to inhibit thrombin (factor IIa) and fibrin formation is reduced such that the correlation between dose and response is lost and increasing the heparin dosage will not result in the desired anticoagulation efect.^5^ It may also cause heparin-induced thrombocytopenia (HIT). The development of HIT is more common in adults, and it is a potentially life-threatening immune-mediated prothrombotic disorder, especially in patients exposed to UFH multiple times.^6, 7^

While UFH requires a sufficient level of AT in the blood to be effective, bivalirudin, a direct thrombin inhibitor, does not require AT to be effective. Bivalirudin has a shorter duration of action (25 minutes) than UFH. Bivalirudin is metabolized mainly by proteolytic enzymes, and 20% is renally excreted. Bivalirudin can be rapidly removed by continuous renal replacement therapy and therapeutic plasma exchange.^8^ Bivalirudin has disadvantages such as the lack of specific antidote, higher cost, and limited ECMO experience.^1^ When using bivalirudin, low-flow areas in the circuit (e.g., laboratory access lines or reperfusion cannulas) may clot and require frequent changes.^9^ Bivalirudin binds directly to thrombin, independent of AT, making it safer in patients with low or fluctuating AT activity.

It also does not bind to other plasma proteins or cells and, as a result, does not cause day-to-day changes in serum chemistry or cell counts. This allows for a more predictable dosing regimen with less bleeding and a consistent anticoagulant effect compared to UNFH.

Finally, it does not cause immune-mediated thrombocytopenia, such as HIT.^10^

Based on the intense working conditions during the coronavirus disease-2019 (COVID-19) period and the need to reduce patient contact, we deemed it appropriate to switch to bivalirudin, which has been shown to have fewer complications and more consistent aPTT monitoring in studies.

The aim of this study was to evaluate the safety, efficacy, and cost-effectiveness of using bivalirudin instead of UFH as the primary anticoagulant in Veno-Venous (V-V) ECMO patients.

## Methods

### Study Design

This study was performed retrospectively on patients hospitalized in the adult intensive care unit of the University of Health Sciences Türkiye, İstanbul Training and Research Hospital between March 31, 2013 and April 31, 2022. This study is a before-after study. Anticoagulant changes were needed due to the increase in patient density, and ECMO-related complications in the COVID-19 pandemic.

The study was conducted in full accordance with local Good Clinical Practice (GCP) guidelines and current legislation. Ethical approval was obtained from the Clinical Research Ethics Committee of the University of Health Sciences Türkiye, Bakırköy Dr. Sadi Konuk Training and Research Hospital (approval no.: 2022-07-09, date: 09.05.2022). Informed consent for the study was obtained from patients or their relatives.

### Study Population and Data Collection

Patients aged >18 years who underwent V-V ECMO for acute respiratory distress syndrome (ARDS) were included in the study population.

Exclusion criteria were as follows: age <18 years, pregnant women, patients who died within the first 48 hours, patients who underwent ECMO for respiratory failure secondary to trauma, patients who were referred after ECMO support was started, patients who underwent ECMO for bridging purposes for lung transplantation, patients who used anticoagulants other than heparin or bivalirudin, patients who used both heparin and bivalirudin at different times (Table 1).   

Study data were obtained retrospectively from the “ImdSoft-Metavision/QlinICU Clinical Decision Support Software” system. Demographic data (gender, age, weight, height, BMI), comorbidity, history of antiaggregant drug use, clinical findings, complications, laboratory, and other data of patients, before and during ECMO, were obtained from the decision support system and recorded. Before ECMO procedure, Sequential Organ Failure Score (SOFA) and Charlson Comorbidity Index (CCI) of the patients were calculated using the available data (Supplementary File 1).

### Study Protocol

The decision for ECMO supportive therapy was made by the ICU ECMO physician team [It was created according to the Extracorporeal Life Support Organization (ELSO) criteria in which our clinic is located].

Routinely, 50-100 IU kg UFH was administered for thrombosis prophylaxis 5 minutes before ECMO vascular cannulation. Routine therapeutic anticoagulant infusion was started after ECMO initiation. For monitoring, only activated partial thromboplastin clotting time (aPTT), which ELSO recommended, was used in all patients. We followed our patients with aPTT because activated clotting time is not licensed for monitoring DTIs, and the results vary depending on many factors, including platelet count and function, fibrinogen level, clotting factor deficiencies, temperature, hemodilution, and technical factors. The therapeutic range for the aPTT was set at 1.5 to 2.5 times the patient’s pretherapy baseline aPTT. To achieve the target aPTT level, measurements were performed every 4-6 hours; the first measurement was calculated within 1 hour after the start of anticoagulant infusion. When the target aPTT range was exceeded, measurements were performed every 2 hours until the normal aPTT target range was achieved after dose adjustment. UFH infusion dose was started as 10-15 units kg^-1^ hr^-1^. The UFH infusion dose was increased by 2-3 units kg^-1^ hr^-1^. In cases where adequate aPTT response could not be obtained despite increasing the UFH infusion dose and 10-15 units kg^-1^ IV bolus administration, FFP (fresh frozen plasma) replacement was performed considering AT deficiency. Bivalirudin infusion dose was increased by 0.01 mg kg^-1^ hr^-1^. In uncontrolled bleeding or bleeding requiring blood replacement, anticoagulant infusion was interrupted until the bleeding was controlled or until the target aPTT level was achieved, until the aPTT was >120 s. The control aPTT was checked every 2 hours. ECMO blood flow was adjusted according to extracorporeal circuit pressures and pre- and post-oxygenation O_2_ content.

Doppler ultrasonography was used to diagnose vascular thrombosis events. Visible thrombi in the oxygenator, pump head, cannulas, or other extracorporeal areas were considered circuit thrombosis. Bleeding from pericanular, pulmonary (intratracheal), urinary tract, gastrointestinal tract, and other areas (mouth, nose, etc.) requiring blood replacement was recorded separately.

Hemogram, biochemistry, arterial blood gas, INR and fibrinogen laboratory tests were checked daily during ECMO. After the start of ECMO, appropriate blood product replacement was performed to regulate hemoglobin concentration (target >7-9 g dL^-1^), platelet count (target >50 10^9^ L, target >100 10^9^ L if active bleeding), fibrinogen level (target >100 mg dL^-1^, active bleeding, target >150 mg dL^-1^) and INR (target <1.5, bleeding <3) levels. Random, pooled random, or apheresis platelet solutions were used to increase platelet levels (target >50x10^9^ L if no active bleeding, target >100x10^9^ L if active bleeding). However, for statistical evaluation, one apheresis platelet solution was considered as 8 random platelet solutions, and one pooled platelet solution was considered as 4 random platelet solutions. When calculating the cost, the total drug doses (daily average) given to the patient during the study period were calculated, including the costs of the blood products given. The cost of laboratory test results was not added. The costs of the study were calculated on the date the data were collected.

### Endpoint

The primary outcome was the statistical difference between the groups receiving UFH and bivalirudin in the effectiveness of anticoagulation. The secondary outcome was the statistical difference in blood product replacement, cost-effectiveness between both groups, and 15-day thrombosis (vascular, ECMO circuit) development and bleeding development.

### Statistical Analysis

Statistical analysis was conducted using IBM SPSS Statistics for Windows, version 25.0 (IBM Corp., Armonk, NY, USA). The Shapiro-Wilk test was used to determine if the data were normally distributed. Categorical variables are given as frequency (n) and percentage (%), while numerical variables are presented as mean ± standard deviation or median with interquartile range. The Independent Samples t-test was used to compare the quantitative variables with normal distribution between the two groups. The Mann-Whitney U test was used for comparisons between two groups of quantitative variables that did not show normal distribution. Pearson chi-square, continuity correction, or Fisher’s exact test was used to compare categorical variables. Statistical significance was accepted as *P *< 0.05.

## Results

A total of 56 patients, 25 receiving UFH and 31 receiving bivaluridine, were enrolled in our study. The demographic data, comorbidities, history of antiaggregant drug use, and SOFA organ failure score of all patients before ECMO are shown in Table 2. Demographic data and SOFA scores indicating disease severity were similar. CCI was higher in the UFH group, which consisted of mixed ECMO patients, whereas all patients in the bivaluridine group had received ECMO for COVID-19 pneumonia.

In the UFH group, the time to reach the target aPTT interval was 6 hours and the percentage of time spent in the target aPTT interval up to 15 days was 61.48%. In the bivalirudin group, the time to reach the target aPTT interval was 9 hours and the percentage of time spent in the target aPTT interval up to 16 days was 62.65% (Table 3). The median total ECMO day was 16 (6.5-24) in the UFH group and 22 (14-42) in the bivaluridine group.

The number of patients with thrombosis, the number of patients with bleeding, the amount of blood product replacement, laboratory parameters, and cost data up to the first 15 days in both patient groups are given in Table 3. In the UFH group, thrombus was observed in ECMO circuit components in a total of 5 patients (20%), but no vascular thrombus was seen. In the bivalirudin group, thrombus was seen in 2 patients with an incidence rate of 6.5% (1 patient in both vascular and ECMO circuits, and 1 patient only in the ECMO circuit). The mean erythrocyte solution replacement (12.04±8.01; 7.9±4.71) and median FFP solution replacement [4 (2-6); 1 (0-4)] were higher in the UFH group than in the bivalirudin group. The mean minimum hemoglobin was lower in the UFH group than in the bivalirudin group (6.71±1.08; 7.68±0.69). The use of UFH for anticoagulation in V-V ECMO patients was much more advantageous in terms of cost-effectiveness compared to the use of bivalirudin (*P* < 0.001).

## Discussion

Anticoagulation in ECMO remains a challenging issue. UFH has become the anticoagulant of choice in ECMO patients due to its ease of monitoring, low cost, and abundance of data supporting its use over other parenteral anticoagulants. Bivalirudin is seen as an anticoagulation agent that can be used to limit the potential side effects of UFH.^9^

The primary outcome of our study was anticoagulation effectiveness, and no significant difference was found between the two groups. There was no statistically significant difference between the UFH and bivalirudin groups in terms of the time to reach the target aPTT level [6 (3.5-11); 9 (4-19)] and the percentage of time elapsed in the target aPTT interval (61.48±14.72; 62.65±11.99). The fact that the patient population using bivaluridine had COVID-19-related ARDS may also have made aPTT regulation difficult. In a recent study, 18% of the UFH cohort switched to the bivalirudin cohort because the therapeutic range could not be reached or maintained, and 20% of 50 patients receiving UFH never reached therapeutic targets during ECMO treatment. Among those who reached the therapeutic range, patients in the UFH cohort required significantly more dose titration and spent less time within the therapeutic range compared with patients receiving bivalirudin.^11^

There are a limited number of retrospective studies with low case volume on the use of bivalirudin as a single anticoagulant in ECMO patients.^12, 13, 14, 15, 16^ However, in these studies, the use of bivalirudin alone was found to be safer than UFH in terms of the development of thrombus and bleeding events. In our study, when the number of patients with thrombus and bleeding and also the total amount of replaced blood products are evaluated together, it may be said that the use of bivaluridine is more suitable than the use of UFH in patients undergoing V-V ECMO. In the bivaluridine group, where all patients had COVID-19, no difference was seen in thrombotic events despite the prothrombotic effect of COVID-19.

Patients on UFH and bivaluridine were compared in terms of the number of patients who developed thrombus or bleeding, and the amount of blood product replacement up to the first 15 days of V-V ECMO initiation. There was no statistically significant difference in the number of patients who developed thrombus in the UFH group [5 (20%); 2 (6.5%); *P*=0.223] or in the number of bleeding events requiring blood product replacement, including pericanular, pulmonary, urinary tract (hematuria), gastrointestinal tract (melena or hematemesis), and other sites (mouth, nose, intracranial, etc.). The total amount of replaced erythrocyte solution was statistically significantly higher in the UFH group than in the bivalirudin group (12.04±8.01, 7.9±4.71, *P*=0.028). The total amount of FFP solution replaced was also significantly higher in the UFH group than in the bivalirudin group [4 (2-6), 1 (0-4), *P*=0.001].

A decrease in all blood cells and coagulation factors due to extracorporeal destruction is expected with ECMO support therapy. In our study, we investigated whether there was any difference in terms of this decrease, in patients using UFH and bivalirudin. Although more erythrocyte replacement was performed in the UFH group, the minimum hemoglobin levels were statistically significantly lower than in the bivalirudin group [6.71±1.08; 7.68±0.69; *P *< 0.001]. In this respect, the data were consistent. In the UFH group, lower values of minimum platelet count could have been expected due to the potential for HIT. However, the prevalence of HIT development in ECMO patients has been found to be between 0.5-5%.^17, 18^ Therefore, there may not have been a statistically significant difference between the minimum platelet levels between both groups.

The fact that the cost of providing 15-day anticoagulation in the UFH group is considerably lower than in the bivaluridine group is an advantage for the use of UFH. There are also studies showing that the total cost of bivalirudin use in ECMO patients is lower, but these studies include pediatric patients, and veno-arterial ECMO patients.^12, 19^ It is clear that the cost of UFH is considerably lower than that of bivalirudin, but the total dose requirement of bivalirudin is fewer in pediatric patients and patients with renal impairment. The number of aPTT measurements also requires additional costs, but this was not calculated in our study.

Since the median V-V ECMO duration of the UFH group was found to be statistically significantly shorter than that of the bivaluridine group, a comparison of the ECMO durations based on the median of 15 days in the UFH group was performed [16 (6.5-24), 22 (14-42), *P*=0.01]. The reason for the higher V-V ECMO duration in the bivaluridine group compared to the UFH group may be because of the introduction of bivaluridine infusion into our routine use since 2020. Additionally, the patient population consists of patients in whom V-V ECMO was started due to respiratory failure caused by COVID-19. As in our study, it is seen that in studies on COVID-19 patients who received ECMO, the total ECMO duration may last longer than in non-COVID patients.^20, 21^

Trigonis et al.^17^ showed no statistically significant difference regarding thrombus, bleeding and in-hospital mortality between COVID and non-COVID patients on ECMO.

### Study Limitations

The main limitations of our study were that it was single-center and retrospective. Although blood product replacement was statistically significant in our study, erythrocyte and FFP transfusion is a practice that varies depending on the physician. Although it was retrospective, the use of the “ImdSoft-Metavision/QlinICU Clinical Decision Support Software” system for the collection of study data was a strength of the study. In addition, since bivalirudin entered our routine use as of 2020, the ECMO etiology of the group using bivalirudin consisted entirely of COVID-19 patients. In contrast, the etiology of ECMO in the UFH group consisted of H1N1 influenza, COVID-19, bacterial pneumonia, and causes secondary to sepsis.

## Conclusion

The use of bivaluridine as a primary anticoagulant does not lead to any decrease in anticoagulant efficacy. The use of bivaluridine alone as the primary anticoagulant in patients on ECMO support therapy does not lead to any increase in the development of thrombus or bleeding events, but may lead to a significant reduction in the need for erythrocyte and FFP replacement.

## Ethics

**Ethics Committee Approval:** Ethical approval was obtained from the Clinical Research Ethics Committee of the University of Health Sciences Türkiye, Bakırköy Dr. Sadi Konuk Training and Research Hospital (approval no.: 2022-07-09, date: 09.05.2022).

**Informed Consent:** Informed consent for the study was obtained from patients or their relatives.

## Supplementary Materials

Supplementary File 1 https://d2v96fxpocvxx.cloudfront.net/df15911b-c92e-4fb1-a3c6-b4a159ab0d2e/content-images/069ff38d-0c12-4ca1-bf9c-96592d23ad0a.pdf

## Figures and Tables

**Table 1. The Study Flowchart:** 

Therapeutic respiratory failure (n = 5)
Ex in 48 hours (n = 4)
<18 years old (n = 1)
Those transferred from external center bt ECMO (n = 1)
ECMO for bridging lung transplantation (n = 3)
Who use partly UFH and bivalurudin (n = 1)
Those using anticoagulants other than UFH or bivaluridin (n = 1)
Pregnancy (n = 1)

UFH, unfractionated heparin; ECMO, extracorporeal membrane oxygenation

**Table 2. Thrombosis, Bleeding, Blood Replacement, and Other Features in All Patients and Patient Groups:** 

**-**	**All patients (n=56)**	**UFH (n=25)**	**Bivaluridin (n=31)**	***P***
Time to aPTT target (hour)	7.5 (4-17.5)	6 (3.5-11)	9 (4-19)	0.287
Percentage of time at aPTT target	62.13±13.16	61.48±14.72	62.65±11.99	0.745
Thrombosis	7 (12.5%)	5 (20%)	2 (6.5%)	0.223
ECMO circuit thrombosis	7 (12.5%)	5 (20%)	2 (6.5%)	0.223
Vascular thrombosis	1 (1.8%)	0 (0%)	1 (3.2%)	1.00
Bleedings	38 (67.9%)	18 (72%)	20 (64.5%)	0.758
Pericanular bleeding	24 (42.9%)	14 (56%)	10 (32%)	0.13
Pulmoner bleeding	22 (39.3%)	11 (44%)	11 (35.5%)	0.709
Hematuria	2 (3.6%)	1 (4%)	1 (3.2%)	1.00
Melena or hematemesis	3 (5.4%)	3 (12%)	0 (0%)	0.083
Other bleedings	8 (14.3%)	3 (12%)	5 (16.11%)	0.720
Erythrocyte replacement	9.75±6.66	12.04±8.01	7.9±4.71	0.028*
FFP replacement	2 (0-5)	4 (2-6)	1 (0-4)	0.001**
Platelet replacement	0 (0-3.75)	0 (0-13)	0 (0-1)	0.393
Minimum hemoglobin (g dL^-1^)	7.25±1	6.71±1.08	7.68±0.69	<0.001**
Maximum INR	1.69 (1.49-2.43)	1.67 (1.34-2.34)	1.75 (1.55-2.46)	0.204
Minimum fibrinogen (mg dL^-1^)	255±102	256±97	255,48±107	0.982
Minimum platelet (10^9^ L)	60 (38.5-88.25)	60 (35-86)	60 (38-95)	0.980
Anticoagulant cost ($)	141.9 (42.3-600.7)	38.1 (13.5-48.7)	463.7 (194.3-819.8)	<0.001**
CRRT	32 (57.1%)	16 (%64)	16 (51.6%)	0.510
Therapeutic plasma exchange	17 (30.4%)	5 (20%)	12 (38.7%)	0.222
Total ECMO days	19 (10.2-33.7)	16 (6.5-24)	22 (14-42)	0.01*
In-hospital mortality	44 (78.6%)	20 (80%)	24 (77.4%)	1.00

*P < 0.05, **P < 0.01.

aPTT, activated partial thromboplastin clotting time; FFP, fresh frozen plasma; CRRT, continue renal replacement treatment; INR, international normalized ratio.

**Table 3. Demographic, Comorbidity and Other Data in All Patients and Patient Groups:** 

**-**	**All patients (n=56)**	**UFH (n=25)**	**Bivaluridin (n=31)**	***P***
Male gender	32 (57.1%)	13 (52%)	19 (61.3%)	0.67
Age	39.91±11.14	41.8±13.33	38.39±8.93	0.279
Body mass index	27.54 (25.42-31.15)	26.23 (24.7-31.2)	28 (26.1-30.1)	0.242
**Comorbidity**				
CCVD	15 (26.8%)	11 (44%)	4 (12.9%)	0.021*
Diabetes mellitus	10 (17.9%)	6 (24%)	4 (12.9%)	0.315
CLD	6 (10.7%)	2 (8%)	4 (12.9%)	0.682
CKD	1 (1.8%)	1 (4%)	0 (0%)	0.446
Other comorbidities	12 (21.4%)	6 (24%)	6 (19.4%)	0.925
CCI	0 (0-2)	1 (0-3)	0 (0-1)	0.002**
Antiaggregant use	4 (7.1%)	2 (8%)	2 (6.5%)	1.00
SOFA score	11.39±3.19	11.2±3.43	11.55±3.04	0.689

*P < 0.05, **P < 0.01.

UFH, unfractionated heparin; CCVH, chronic cardiovascular disease; CLD, chronic lung disease; CKD, chronic kidney disease; CCI, Charlson Comorbidity Index; SOFA, Sequential Organ Failure Assessment.
